# Toward personalized prediction: a multicenter machine learning model for omalizumab response duration in moderate-to-severe perennial allergic rhinitis

**DOI:** 10.3389/falgy.2026.1784032

**Published:** 2026-04-10

**Authors:** Ziqi Miao, Lin Dong, Jin Liu, Le Li, Xiang Dong, Shuchen Zhang, Rongfei Zhu, Yuqin Deng

**Affiliations:** 1 Department of Otolaryngology-Head and Neck Surgery, Renmin Hospital of Wuhan University, Wuhan, Hubei, China; 2 Department of Allergy, Tongji Hospital, Tongji Medical College, Huazhong University of Science and Technology, Wuhan, Hubei, China; 3 Department of Allergology, Zhongnan Hospital of Wuhan University, Wuhan, Hubei, China

**Keywords:** machine learning, moderate-to-severe perennial allergic rhinitis, multicenter study, omalizumab, personalized prediction

## Abstract

**Background:**

Omalizumab effectively improves quality of life in patients with moderate-to-severe perennial allergic rhinitis (PAR) uncontrolled by conventional medications. However, the duration of its efficacy remains unclear, and there is a lack of effective tools for individualized prediction.

**Objective:**

This study aimed to identify predictors of Omalizumab duration of efficacy in moderate-to-severe PAR patients, then develop and validate an interpretable, machine learning-based predictive model to forecast the duration of efficacy following treatment.

**Methods:**

This multicenter retrospective study included 561 patients with moderate-to-severe PAR treated with Omalizumab at three clinical institutions. The trial was registered at Chinese Clinical Trial Registry, ChiCTR2500112034. Patient characteristics included age, sex, serum total IgE concentration, serum specific IgE (sIgE) concentrations including *Dermatophagoides pteronyssinus* (D1) and *Dermatophagoides farina* (D2), comorbid conditions (asthma, urticaria, conjunctivitis, atopic dermatitis), and injection frequency. Univariate and multivariate Cox regression were employed to investigate independent predictors of Omalizumab efficacy, with restricted cubic splines for dose-response analysis. Five survival machine learning models (CPH, XGBoost, RSF, SSVM, CoxBoost) were constructed and compared by comprehensive metrics. The optimal model was interpreted using the SHapley Additive exPlanations (SHAP) and deployed as an online web application.

**Results:**

Univariate Cox regression identified age, D1, D2, asthma and injection frequency as independent factors affecting Omalizumab efficacy duration. Multivariate analysis did not confirm D2 as significant. Dose-response analysis demonstrated enhanced protective effects beyond four injections. Among the five models, RSF demonstrated robust predictive performance. SHAP analysis identified injection frequency, age, D1, D2, and coexisting asthma as the most critical factors.

**Conclusion:**

This study developed and validated a machine learning-based model capable of forecasting the duration of Omalizumab efficacy in moderate-to-severe PAR based on readily available clinical variables.

## Graphical Abstract

**Figure:**
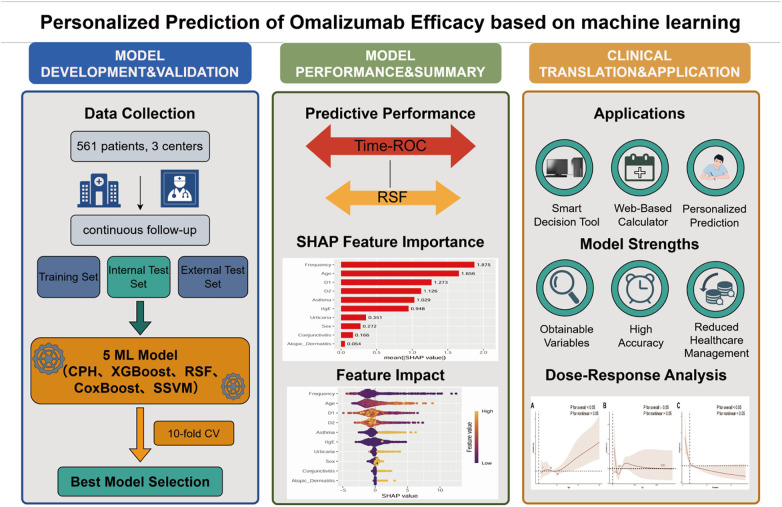
Workflow chart. A total of 561 patients with moderate-to-severe PAR treated with Omalizumab were included, experienced doctors conducted continuous follow-up on these patients and collected clinical data. including demographic data, laboratory data, comorbidities and injection frequency. All data were divided into a training set, an internal test set and two external test sets. Five survival machine learning models (CPH, XGBoost, RSF, CoxBoost, SSVM) were constructed and compared. They were optimized using repeated ten-fold cross-validation, and these models were compared through multiple complementary metrics. The robotic model was interpreted using the SHAP method, and then was deployed on an online web application. Dose- response analysis of numerical variables was conducted using restrictive cubic splines. PAR, perennial allergic rhinitis; CPH, cox proportional hazards model; XGBoost, eXtreme gradient boosting; RSF, random survival forests; SSVM, survival support vector machine; CoxBoost, cox regression with boosting; SHAP, SHapley additive exPlanations.

## 1 Introduction

Allergic rhinitis (AR) is a chronic, non-infectious inflammatory condition primarily mediated by immunoglobulin E (IgE) following exposure to allergens. It affects approximately 14% of the U.S. population, with prevalence rates reaching 20.9% in China and 20% in Canada (1, 2). The disease burden extends beyond high prevalence to include impaired quality of life—sleep disturbances, reduced productivity—and increased healthcare resource utilization (3, 4). Current treatment strategies include environmental control, pharmacological interventions, and allergen-specific immunotherapy. Although standard pharmacological management—such as intranasal/oral corticosteroids, antihistamines, and leukotriene receptor antagonists—can alleviate symptoms, their effectiveness varies widely among patients. A significant subset of individuals does not achieve satisfactory outcomes, which markedly impairs their quality of life and highlights a critical unmet need for more effective therapeutic options (5, 6).

In this context, the international consensus by Wise et al. suggested Omalizumab for patients with moderate-to-severe AR who remain poorly controlled despite optimal conventional treatment and allergen avoidance (7). Omalizumab, a humanized anti-IgE monoclonal antibody, works by binding to free IgE and preventing its interaction with high-affinity (Fc*ε*RI) and low-affinity (CD23) receptors on mast cells, basophils, and B cells (8). This mechanism not only blocks degranulation and release of allergic mediators but also downregulates Fc*ε*RI receptors on effector cells and may modulate IgE-mediated antigen presentation (9). While the U.S. Food and Drug Administration (FDA) has approved Omalizumab for chronic spontaneous urticaria (CSU), severe asthma, and food allergy, its use in AR remains off-label in many regions.

Nevertheless, a growing body of evidence—from randomized controlled trials to real-world studies—has demonstrated its efficacy and safety in AR (10–12), with systematic reviews confirming improvements in nasal symptoms, reduced need for rescue medication, and enhanced quality of life (13). Among the emerging biologic therapies, Omalizumab holds a unique position. Other biologics, such as Dupilumab, Mepolizumab, Benralizumab and Tezepelumab, primarily target downstream type 2 inflammatory pathways (e.g., IL-4/13, IL-5 or TSLP) (14). In contrast, Omalizumab directly targets IgE, the primary trigger of the allergic cascade in AR. Furthermore, the extensive real-world evidence for Omalizumab provides a robust foundation for investigating its long-term efficacy. Clinical use of Omalizumab has proven effective in controlling symptoms; however, factors affecting the duration of its efficacy after treatment completion remain uncertain.

Although numerous studies have investigated biomarkers affecting the efficacy of Omalizumab, several limitations remain. Most studies have focused on CSU or asthma rather than AR (15–18). Among the biomarkers proposed, clinical relevance remains contentious due to conflicting results and practical barriers to obtaining certain markers in routine outpatient settings (19–21). Moreover, existing AR research has concentrated on predicting treatment response itself, without addressing the duration of effect after treatment stops (22). Finally, machine learning approaches to predict Omalizumab outcomes remain nascent—current efforts have focused almost exclusively on forecasting IgE concentrations (23), and limited established predictive models exist for predicting treatment duration in AR.

In this study, we aimed to develop and validate an interpretable machine learning model using multicenter data to predict how long Omalizumab remains effective in patients with moderate-to-severe perennial allergic rhinitis (PAR) triggered by sensitization to *Dermatophagoides pteronyssinus* (D1) or *Dermatophagoides farina* (D2). Using SHapley Additive exPlanations (SHAP), we quantified the contribution of individual factors and enhanced model interpretability. The resulting model was deployed as a user-friendly web interface to facilitate clinical application.

## 2 Materials and methods

### 2.1 Data collection

This study utilized three independent datasets to develop and validate interpretable machine learning models. The development set comprised 435 patients with moderate-to-severe PAR who received anti-IgE therapy at the Otolaryngology outpatient clinic of Renmin Hospital of Wuhan University between July 2020 and August 2024. Two external test sets were obtained from other institutions: external test set 1 included 74 patients from the Allergy Department at Tongji Hospital (November 2020 to March 2024), and external test set 2 included 78 patients from the Allergy Department at Zhongnan Hospital of Wuhan University (May 2018 to December 2024).

Patients were eligible for inclusion if they met all of the following criteria: (1) aged 6 to 65 years; (2) diagnosed with AR by specialist physicians according to current diagnostic guidelines (24); (3) experienced typical PAR symptoms without seasonal aggravation for more than one year; (4) demonstrated sensitization to D1 or D2, defined as a skin prick test reaction of ≥ ++ or serum specific IgE (sIgE) ≥ 0.7 kUA/L; (5) had serum total IgE (tIgE) levels between 60 and 3000 kU/L; (6) presented with clinically relevant allergic symptoms triggered by D1 or D2 exposure; (7) had a total nasal symptom score (TNSS) ≥ 6 despite monotherapy or combination therapy with oral antihistamines, intranasal corticosteroids, and/or leukotriene receptor antagonists; (8) experienced impaired quality of life (e.g., sleep disturbances, reduced daily/leisure activities, impaired school or work performance, or bothersome symptoms); and (9) received anti-IgE treatment and provided signed informed consent for the treatment protocol.

Patients were excluded if they met any of the following: (1) history of AR with seasonal exacerbations; (2) positive results from skin prick test or serum sIgE to any of the following pollen allergens: *Betula verrucosa* (t3), *Cupressus sempervirens* (t6), *Platanus acerifolia* (t11), Tree mix 5 (tx5), Tree mix 20 (tx20), *Humulus lupulus* (w22), *Artemisia vulgaris* (w6), *Ambrosia artemisiifolia* (w1), *Chenopodium album* (w10), *Xanthium commune* (w13), Weed mix 7 (wx7), Weed mix 5 (wx5), or other relevant pollen allergens; (3) receipt of sublingual or subcutaneous immunotherapy before, during, or after Omalizumab treatment; (4) treatment with oral corticosteroids within 4 weeks prior to initiation of Omalizumab; (5) acute or chronic rhinosinusitis (with or without nasal polyps); (6) hepatic or renal impairment, or pregnancy; (7) acute asthma exacerbation; (8) severe systemic diseases (e.g., immunodeficiency disorders, malignancies); (9) acute respiratory tract infection; or (10) loss to follow-up (21 cases in the development set and 5 cases in external test set 2).

Ultimately, data from 414 patients in the development set, 74 patients in external test set 1, and 73 patients in external test set 2 were included in the final analysis. This retrospective study used anonymized patient data, waiving the requirement for individual informed consent. The study protocol was registered with the Chinese Clinical Trial Registry (ChiCTR2500112034), adhered to the principles of the Declaration of Helsinki, and was approved by the Ethics Committee of Renmin Hospital of Wuhan University [Approval No. 2025K-K101(C01)]. The reporting follows the Transparent Reporting of a multivariable prediction model for Individual Prognosis Or Diagnosis (TRIPOD) statement.

### 2.2 Study variables

Clinical data were extracted from the electronic medical record system (EMRS), including demographic characteristics (age and sex), laboratory parameters (serum tIgE and sIgE levels for D1 or D2), comorbidities (asthma, urticaria, conjunctivitis, and atopic dermatitis), and treatment-related information (frequency of Omalizumab injections). Serum IgE concentrations were measured using the ImmunoCAP system (Thermo Fisher Scientific).

### 2.3 Efficacy assessment

Patients enrolled underwent continuous follow-up to assess disease activity, quality of life, and disease control. A combination of the minimal clinically important difference (MCID) and a validated control threshold was used to ensure clinically meaningful efficacy judgments. Specially, disease activity was evaluated and monitored using the visual analogue scale (VAS) and TNSS. The rhinoconjunctivitis quality of life questionnaire (RQLQ) was employed to assess patients’ quality of life. The combined symptom and medication score (CSMS) and allergic rhinitis control test (ARCT) were used to evaluate and monitor PAR disease control. Among these tools, VAS, TNSS, RQLQ, and CSMS were used in conjunction with MCID to determine efficacy changes, while ARCT adopted an absolute control threshold.

#### 2.3.1 VAS

The VAS is determined by patients marking a point on a 0–10 cm scale corresponding to their symptom severity, with scores ranging from 0 to 10. A score of “0” indicates no symptoms, while “10” represents the most severe symptoms.

#### 2.3.2 TNSS

The TNSS comprises four items (rhinorrhea, nasal congestion, nasal itching, sneezing), each scored on a 0–3 scale where 3 indicates the most severe symptom. The total score is 12 points, primarily used to assess the overall severity of nasal symptoms.

#### 2.3.3 RQLQ

The standard version of the RQLQ comprises 28 items across seven domains: nasal problems, eye problems, activities, sleep, practical problems, non-nose/eye symptoms, and emotional function. Each RQLQ item is scored on a 0–6 scale, with the total RQLQ score being the average of all items. Higher scores indicate poorer quality of life.

#### 2.3.4 CSMS

The CSMS integrates the daily symptom score (dSS) and daily medication score (dMS). The dSS quantifies the severity of six nasal and ocular symptoms ranging from 0 to 3 points. While the dMS provides a standardized assessment of the intensity of rescue medication use, with higher scores assigned to more potent anti-allergic interventions: a score of 0 indicates no medication use; a score of 1 is assigned for oral or ocular antihistamines; a score of 2 corresponds to intranasal corticosteroids (used in conjunction with antihistamines); and a score of 3 represents oral corticosteroids (added to antihistamines and intranasal corticosteroids), yielding a maximum daily score of 3. The final score is the sum of dSS and dMS, ranging from 0 to 6 points. A lower score indicates better symptom and medication control.

#### 2.3.5 ARCT

The ARCT assesses symptom frequency and severity, medication use, and impact on quality of life through five questions, each scored out of 5 points. The ARCT yields a total score ranging from 5 to 25, with higher scores indicating better disease control and a score of 20 or higher validated as the threshold for well-controlled AR (25).

With reference to existing literature and clinical experience, we defined the following criteria: (1)VAS increased of ≥2.0 points compared to the lowest recorded value during the treatment period (26); (2) An increase in TNSS of ≥0.55 points compared to the lowest recorded value during the treatment period (27); (3) An increase in RQLQ of ≥0.22 points compared to the lowest recorded value during the treatment period (28); (4) CSMS increased by ≥0.22 points compared to the lowest recorded value during the treatment period (29); (5) An ARCT total score < 20 points (25). During follow-up, if any of the above conditions occurred, Omalizumab was considered to have lost efficacy, and the time of symptom recurrence was recorded. When conflicting results arose from different indicators, the final determination was based on the comprehensive assessment of patient's chief complaint and the judgment of senior clinicians (with over 15 years of experience).

### 2.4 Data preprocessing

Missing tIgE values were recorded in 10 patients in the development set and 2 patients in external test set 1. These missing data were handled using multiple imputation with the predictive mean matching to reduce bias and ensure robustness in subsequent analyses (30). Serum sIgE concentrations for D1 and D2 were categorized into six levels: level 1 (0.35–0.70 kUA/L), level 2 (0.70–3.50 kUA/L), level 3 (3.50–17.50 kUA/L), level 4 (17.50–50.00 kUA/L), level 5 (50.00–100.00 kUA/L), and level 6 (>100 kUA/L). Comorbidities were binary-encoded.

For model development and internal validation, the development set was randomly partitioned into training and internal test sets at a 7:3 ratio, yielding 290 and 124 cases, respectively (31). Random assignment ensured comparable distributions of key variables between the two subsets. Two independent external datasets from other hospitals were used for external validation to assess model generalizability.

### 2.5 Univariate and multivariate cox regression analysis

To investigate independent factors affecting the efficacy duration of Omalizumab treatment for moderate-to-severe PAR, we conducted univariate and multivariate Cox regression analysis.

### 2.6 Model construction

Five survival analysis methods were employed for model construction: Cox Proportional Hazards Model (CPH), eXtreme Gradient Boosting (XGBoost), Random Survival Forests (RSF), Survival Support Vector Machine (SSVM) and Cox Regression with Boosting (CoxBoost). These methods are well suited for time-to-event analysis and right-censored data. CPH served as a conventional interpretable algorithm, while the machine learning approaches (RSF, XGBoost, SSVM, and CoxBoost) were selected to capture nonlinear relationships, complex interactions, and high-dimensional features without restrictive parametric assumptions (32, 33). To optimize predictive models, ten-fold cross-validation was repeated 400 times in conjunction with hyperparameter grid search on the optimal feature subset. The final hyperparameters were determined based on cross-validation performance, and each model was subsequently refitted using the optimal feature subset and selected hyperparameters.

### 2.7 Model performance comparison and optimization

Model performance was assessed using multiple complementary metrics. Time-dependent receiver operating characteristic (Time-ROC) curves were constructed to evaluate discriminative ability at 3, 6, and 12 months, and the concordance index (C-index) was used to quantify overall discriminative capacity. The 12-month F1 score assessed classification performance, the 12-month Brier score measured prediction accuracy, and the calibration curve slope evaluated agreement between predicted probabilities and observed outcomes. The optimal model was selected based on its consistent performance across the training, internal test, and external test sets.

To enable unified visual comparison in a heatmap, the Brier score (where lower is better) was transformed into a “higher is better” scale as 1−Brier. The calibration slope, ideally equal to 1, was transformed into a calibration index defined as 1−∣slope−1∣.

Model optimization was performed via grid search based on Out-of-Bag (OOB) error to select the optimal number of nodes and splitting method for enhanced predictive accuracy.

### 2.8 Model interpretation and application

In this study, SHAP was employed to interpret model predictions by quantifying each feature's contribution, overcoming the “black box” limitation and providing both local and global explanations (34).

To enhance clinical utility of this study, the final model was deployed as a Shiny-based web application. By inputting relevant clinical information, the platform estimates the probability of Omalizumab efficacy persisting at 3, 6, and 12 months. To evaluate the model's practical application, thirty medical experts with varying experience levels assessed the model's accuracy, portability, interpretability, computational speed, and system stability.

### 2.9 Dose-response relationship

Restricted cubic spline (RCS) regression was used to quantitatively evaluate the dose–response relationship between numerical variables (age, tIgE, injection frequency) and clinical outcomes, with statistical inflection points identified from the fitted spline curves.

### 2.10 Statistical analysis

The sample size for developing this survival prediction model was calculated following the criteria proposed by Riley et al. for time-to-event outcomes (35).

Continuous variables, which did not satisfy normal distribution, were expressed as medians with interquartile ranges and compared using the Mann–Whitney *U*-test. Categorical variables were presented as percentages and compared using the chi-square test where applicable; otherwise, Fisher's exact test or Monte Carlo methods were employed. A two-tailed *P* value < 0.05 was considered statistically significant.

All predictive models were constructed using R software (version 4.4.3). CPH was built with the coxph function (survival package), XGBoost with the xgboost package, RSF with the rfsrc function (randomForestSRC package), SSVM with the survivalSVM function (survivalSVM package), and CoxBoost with the CoxBoost function (CoxBoost package). Hyperparameter grid search was performed using the caret package to determine optimal parameters for each model.

Model performance metrics were calculated as follows: Time-ROC curves were generated using the Time-ROC and ggplot2 packages; the C-index was obtained via the concordance function (survival package); the 12-month F1 score was derived by dichotomizing predicted survival probabilities at a 0.5 threshold and comparing with observed event status; the 12-month Brier score was calculated using the sbrier function (survcomp package); and the calibration curve slope was derived from survfit (survival package) and the base R lm function.

Similarly, RCS function was performed in R (version 4.4.3) to examine dose-response relationships between numerical variables and clinical outcomes.

## 3 Results

### 3.1 Patients baseline analysis

Table 1 summaries the demographic and clinical information of enrolled patients. No significant differences were observed in the distribution of sex, tIgE, atopic dermatitis, symptomatic medication use, and baseline scores between the training set, internal test set, and external test sets (*P* > 0.05). However, notable differences in distribution were evident across certain clinical characteristics among these datasets. For instance, in external test set 1, patients treated with Omalizumab were older, more frequently presented with mild D1 or D2 severity, and had a higher proportion of concomitant urticaria (32.4%). In external test set 2, patients treated with Omalizumab were younger, had a higher proportion of concomitant asthma and conjunctivitis (57.5% and 26.0%, respectively), and received more Omalizumab injections. During follow-up, we observed a significant reduction in CSMS scores in each set after treatment with Omalizumab, as shown in Supplementary Figure S1.

**Table 1:** Comparison of demographic characteristics and clinical characteristics between training set, internal test set, external test set 1 and external test set 2.

Characteristics	Training set (*n* = 290)	Internal test set (*n* = 124)	External test set 1 (*n* = 74)	External test set 2 (*n* = 73)	Overall (*n* = 561)	*P* value
Sex, *n* (%)						0.036
Female	116 (40.0%)	58 (46.8%)	42 (56.8%)	27 (37.0%)	243 (43.3%)	
Male	174 (60.0%)	66 (53.2%)	32 (43.2%)	46 (63.0%)	318 (56.7%)	
Age, years, median (IQR)	14 (9,29)	17 (9,35.5)	32.5 (17.8,42.3)	11 (7,31.5)	16 (9,33.5)	<0.001
sIgE concentrations, kUA/L, *n* (%)						
D1						<0.001
<17.5	42 (14.5%)	22 (17.7%)	29 (39.2%)	12 (16.4%)	105 (18.7%)	
≥17.5	248 (85.5%)	102 (82.3%)	45 (60.8%)	61 (83.6%)	456 (81.3%)	
D2						<0.001
<17.5	41 (14.1%)	21 (16.9%)	27 (36.5%)	10 (13.7%)	99 (17.6%)	
≥17.5	249 (85.9%)	103 (83.1%)	47 (63.5%)	63 (86.3%)	462 (82.4%)	
tIgE concentrations, kU/L, median (IQR)	426.5 (232.8,876)	396.5 (221,726.3)	368.5 (206.5,655.3)	411 (201,851)	403 (226.5,787)	0.417
Comorbidities, *n* (%)						
Asthma	41 (14.1%)	22 (17.7%)	33 (44.6%)	42 (57.5%)	138 (24.6%)	<0.001
Urticaria	47 (16.2%)	17 (13.7%)	24 (32.4%)	7 (9.6%)	95 (16.9%)	<0.001
Conjunctivitis	27 (9.3%)	6 (4.8%)	3 (4.1%)	19 (26.0%)	55 (9.8%)	<0.001
Atopic Dermatitis	4 (1.4%)	1 (0.8%)	2 (2.7%)	4 (5.4%)	11 (2.0%)	0.098
Injection Frequency, median (IQR)	3 (3,4)	3 (3,5)	5 (3,7)	7 (4,13)	4 (3,6)	<0.001
Receipt of symptomatic treatment, *n* (%)	290 (100%)	124 (100%)	74 (100%)	73 (100%)	561 (100%)	
Baseline Score, median (IQR)						
VAS	7 (6.75,8)	7 (7,8)	8 (6.75,9)	8 (7,9)	8 (7,8)	0.343
TNSS	8 (7,10)	8.5 (7,10)	8 (6,9)	8 (7,10)	8 (7,10)	0.271
RQLQ	3 (2,4)	3 (3,5)	4 (3,5)	4 (2.5,5)	3 (2,4)	0.727
CSMS	3 (2,3)	3 (2,3)	3 (2,3)	3 (2,3)	3(2,3)	0.954
ARCT	11(9,13)	11(9,12.75)	10(9,12)	11(10,13)	11(9,13)	0.124

sIgE, specific IgE; D1, *D. pteronyssinus*; D2, *D. farinae*; tIgE, total IgE; VAS, visual analogue scale; TNSS, total nasal symptom score; RQLQ, rhinoconjunctivitis quality of life questionnaire; CSMS, combined symptom and medication score; ARCT, allergic rhinitis control test.

### 3.2 Univariate and multivariate cox regression analysis

We investigated independent factors affecting the duration of Omalizumab treatment efficacy for moderate-to-severe PAR across the entire cohort. Univariate Cox regression analysis identified two potential risk factors (HR > 1, *P* < 0.05): age and the presence of concomitant asthma. D1 and D2 concentrations, along with the frequency of injections, were significant protective factors (HR < 1, *P* < 0.05, Supplementary Table S1). Supplementary Figure S2 displays the forest plot from the multivariable Cox regression analysis, where age, D1 concentration, presence of concomitant asthma, and frequency of injections were significant features (*P* < 0.05).

### 3.3 Model construction and comparison

**Figure 1:**
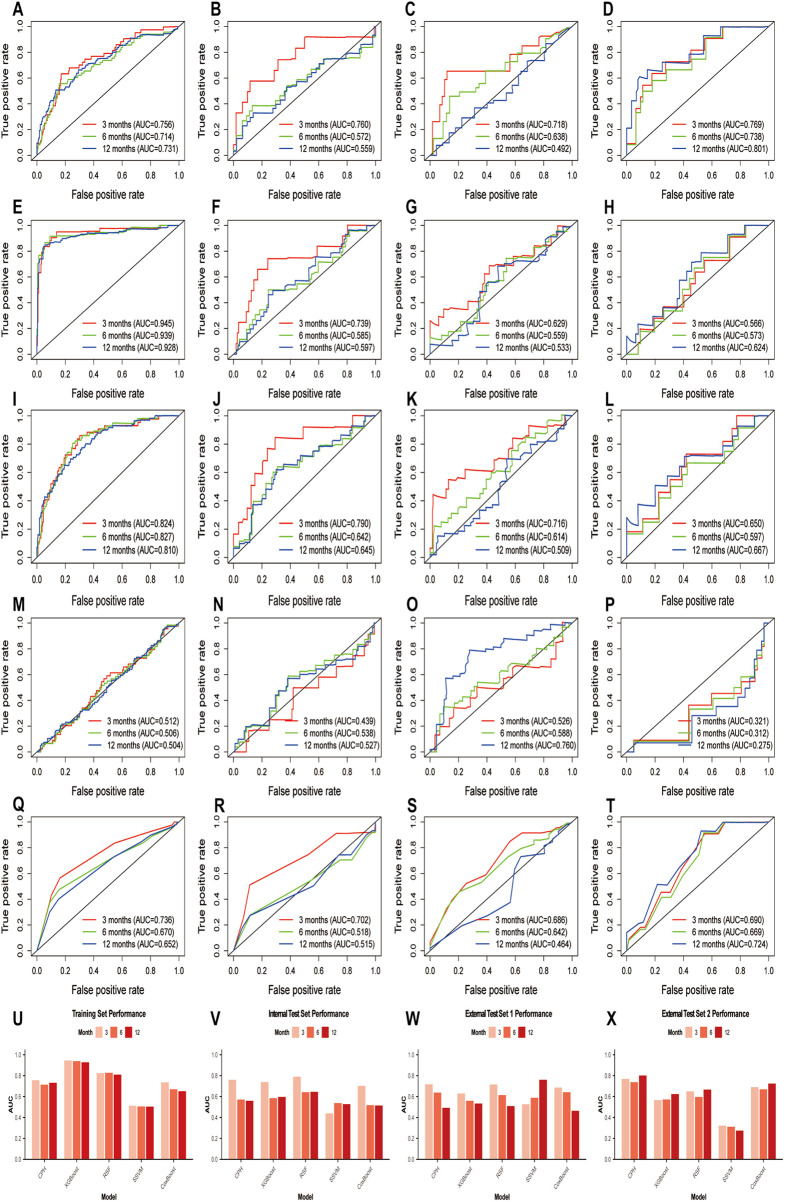
Time-ROC curve of the five machine learning models in predicting the duration of omalizumab efficacy in patients with moderate-to-severe PAR across the training and test sets. Time-ROC curves were shown for the CPH **(A–D)**, XGBoost **(E–H)**, RSF **(I–L)**, SSVM **(M–P)**, and CoxBoost **(Q–T)** models. For each model, the curves are shown for the training set, internal test set, external test set 1 and external test set 2. The area under the curve (AUC) values for these sets were presented as bar plots **(U–X)**.

We performed 400 iterations of ten-fold cross-validation on the training set to construct five models. And the comparison of the accuracy among five models based on Time-ROC was shown in Figure 1.

On the training set, XGBoost demonstrated the highest performance (3-month AUC = 0.945, 6-month AUC = 0.939, 12-month AUC = 0.928, Figure 1E), indicating optimal fit to training data with robust predictive accuracy. RSF followed closely (3-month AUC = 0.824, 6-month AUC = 0.827, 12-month AUC = 0.810, Figure 1I), achieving a high level of performance.

On the internal test set, RSF achieved the highest AUC (3-month AUC = 0.790, 6-month AUC = 0.642, 12-month AUC = 0.645, Figure 1J).

Predictive accuracy varied across time periods on the external test sets. On external test set 1, CPH achieved the highest accuracy for 3 month (3-month AUC = 0.718, Figure 1C), followed by RSF (3-month AUC = 0.716, Figure 1K); at 6-month, CoxBoost yielded the highest AUC (6-month AUC = 0.642, Figure 1S), while SSVM achieved the highest AUC at 12-month (12-month AUC = 0.760, Figure 1O). On external test set 2, CPH achieved the highest prediction accuracy across all time points (3-month AUC = 0.769, 6-month AUC = 0.738, 12-month AUC = 0.801, Figure 1D).

**Figure 2:**
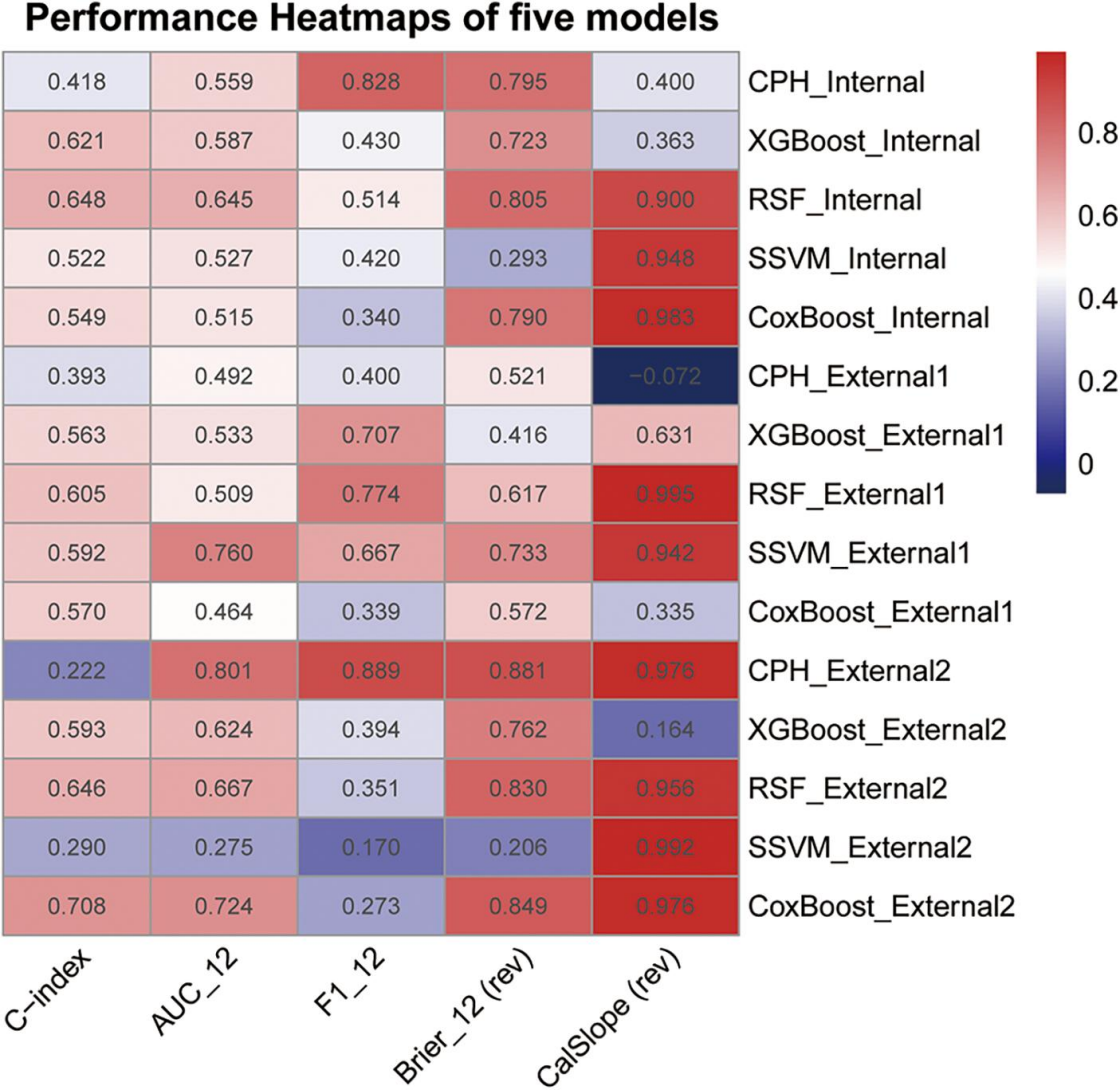
The performance heatmap of five models in predicting the duration of omalizumab efficacy in patients with moderate-to-severe PAR across the training and test sets. The 12-month brier score and calibration slope were presented after transformation (higher is better). CalSlope: the calibration slope.

Figure 2 displays the performance heatmap of five models across the test sets. In the heatmap, C-index, AUC at 12 months, and 12-month F1 score were shown as original values, while the 12-month brier score and calibration slope were presented after transformation (higher is better). RSF consistently exhibited high values across most metrics, whereas SSVM showed poor performance on the internal test and the external set 2.

In summary, considering performance across both training and test sets, we selected RSF as the most suitable model for predicting the efficacy of Omalizumab in moderate-to-severe PAR.

### 3.4 Model optimization

When rebuilding the RSF model on the entire dataset, results showed AUC values exceeding 0.8 for 3-month, 6-month and 12-month (Supplementary Figure S3A), indicating robust model performance. Optimization was conducted using grid search based on OOB error to identify optimal splitting strategies and node counts. Following optimization, the predicted AUC exceeded 0.9 for all observed months (Supplementary Figure S3B), with the highest AUC value recorded for 12-month (12-month AUC = 0.968).

### 3.5 Model interpretation

**Figure 3:**
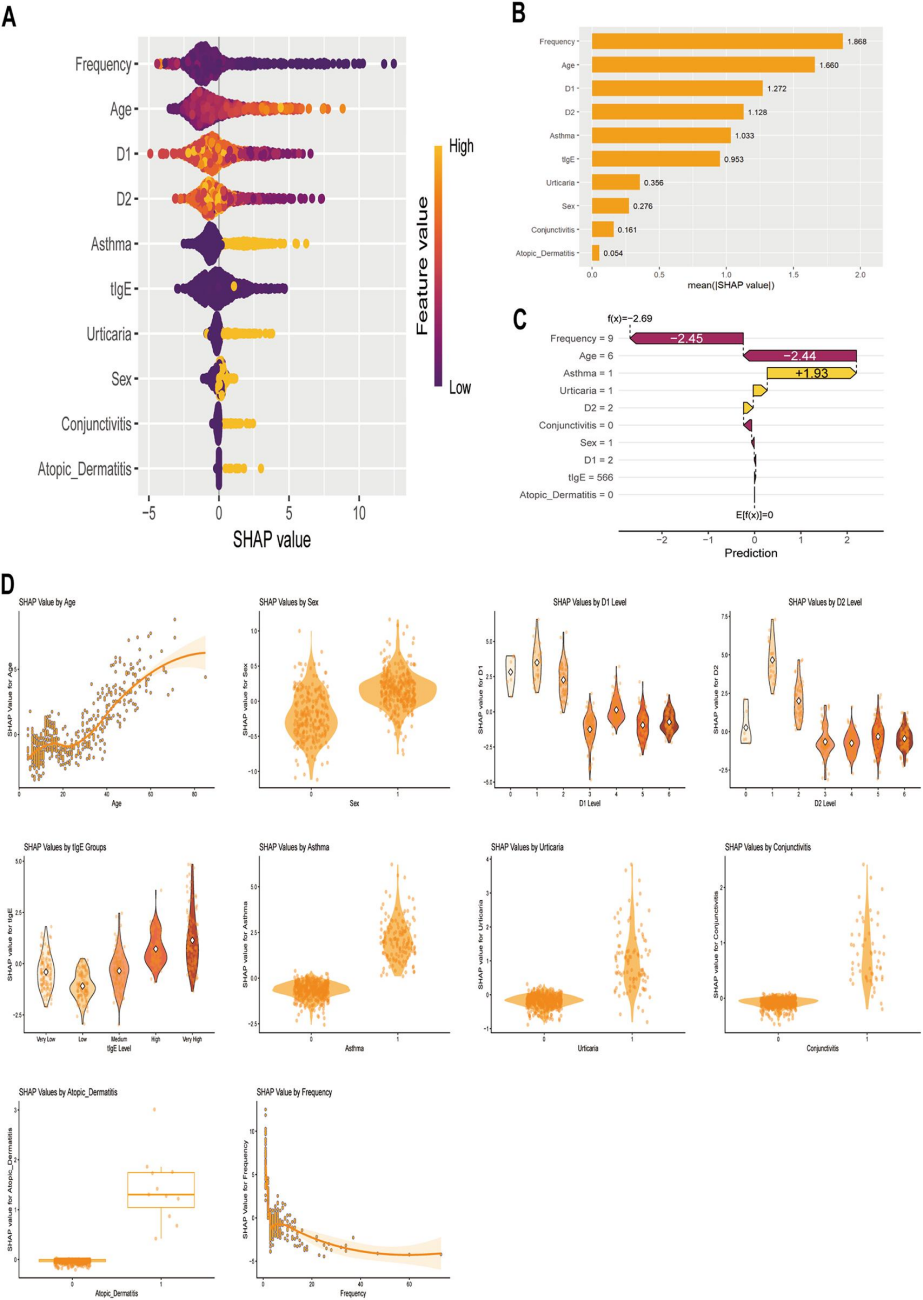
Global and local interpretations of SHAP methods. **(A)** SHAP summary honeycomb plot. The probability of symptoms recurrence increases with the SHAP values of the features. Each dot represents a patient's SHAP value for a given feature, with orange indicating higher feature values and purple indicating lower values. **(B)** SHAP summary bar plot. This plot evaluates the contribution of each feature to the model using mean SHAP values, displayed in descending order. **(C)** SHAP waterfall plot for the fiftieth moderate-to-severe PAR patient. **(D)** SHAP dependence plot. Each dependence plot shows how a single feature affects the model's output, with each point representing a patient. SHAP values are on the *y*-axis, and actual feature values are on the *x*-axis. Features with SHAP values above zero indicated a higher risk of shortening the Omalizumab efficacy duration. D1, *D. pteronyssinus*; D2, *D. farina*; tIgE: total IgE.

To enhance interpretability, SHAP was applied to quantify each variable's contribution to model predictions, providing both global and local explanations. The RSF model global interpretation via a honeycomb plot (Figure 3A) demonstrated the strength and direction of each variable in the prediction process. For instance, fewer injections, older age, lower D1 and D2 concentrations, and comorbid asthma were not conducive to disease control, thereby shortening Omalizumab efficacy duration. Additionally, Figure 3B evaluated the importance of each variable for predicting the outcome based on its average SHAP value, with results ranked in descending order. At the individual level, the SHAP waterfall plot (Figure 3C) illustrated feature contributions for a specific patient (the 50th case). Injection frequency and age showed strong negative contributions (−2.45 and −2.44, respectively), while comorbid asthma contributed positively (1.93), demonstrating how individual features shaped the prediction. Figure 3D compared actual vs. SHAP values for all ten features; features with positive SHAP values (indicating risk factors) were associated with shorter predicted efficacy duration.

### 3.6 Personalized prediction

**Figure 4:**
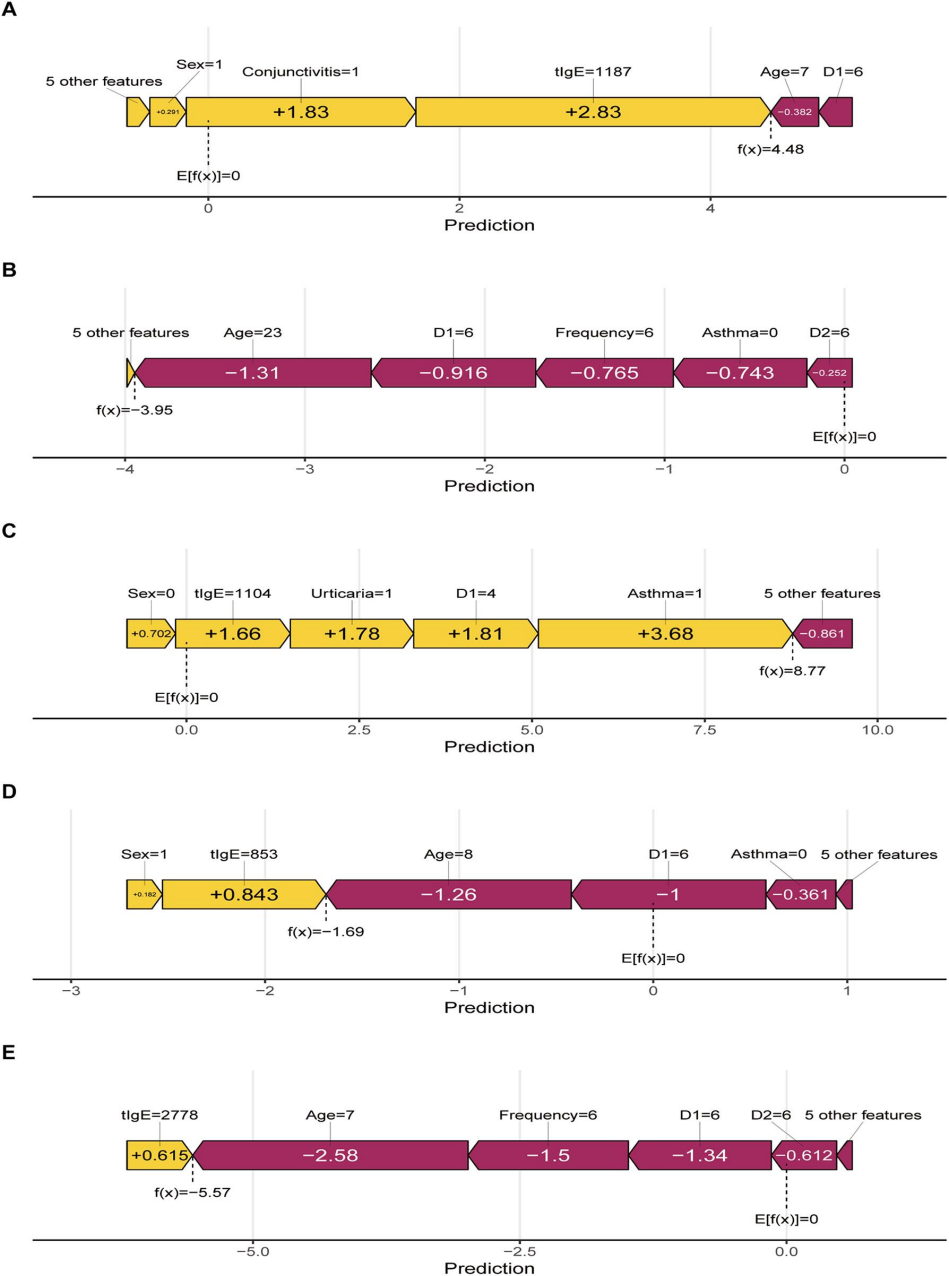
Personalized SHAP analysis of 5 examples (**A-E**) with similar baseline clinical characteristics. D1, *D. pteronyssinus*; D2, *D. farina*; tIgE: total IgE.

To assess the model's ability to provide personalized explanations, five patients with moderate-to-severe PAR and similar baseline characteristics were selected for SHAP analysis, focusing on the top five predictive variables (full patient characteristics were provided in Supplementary Table S2). Despite comparable clinical profiles, these patients exhibited distinct SHAP contribution patterns (Figure 4), demonstrating the model's capacity to generate individualized predictions and explanations.

### 3.7 Model application

**Figure 5:**
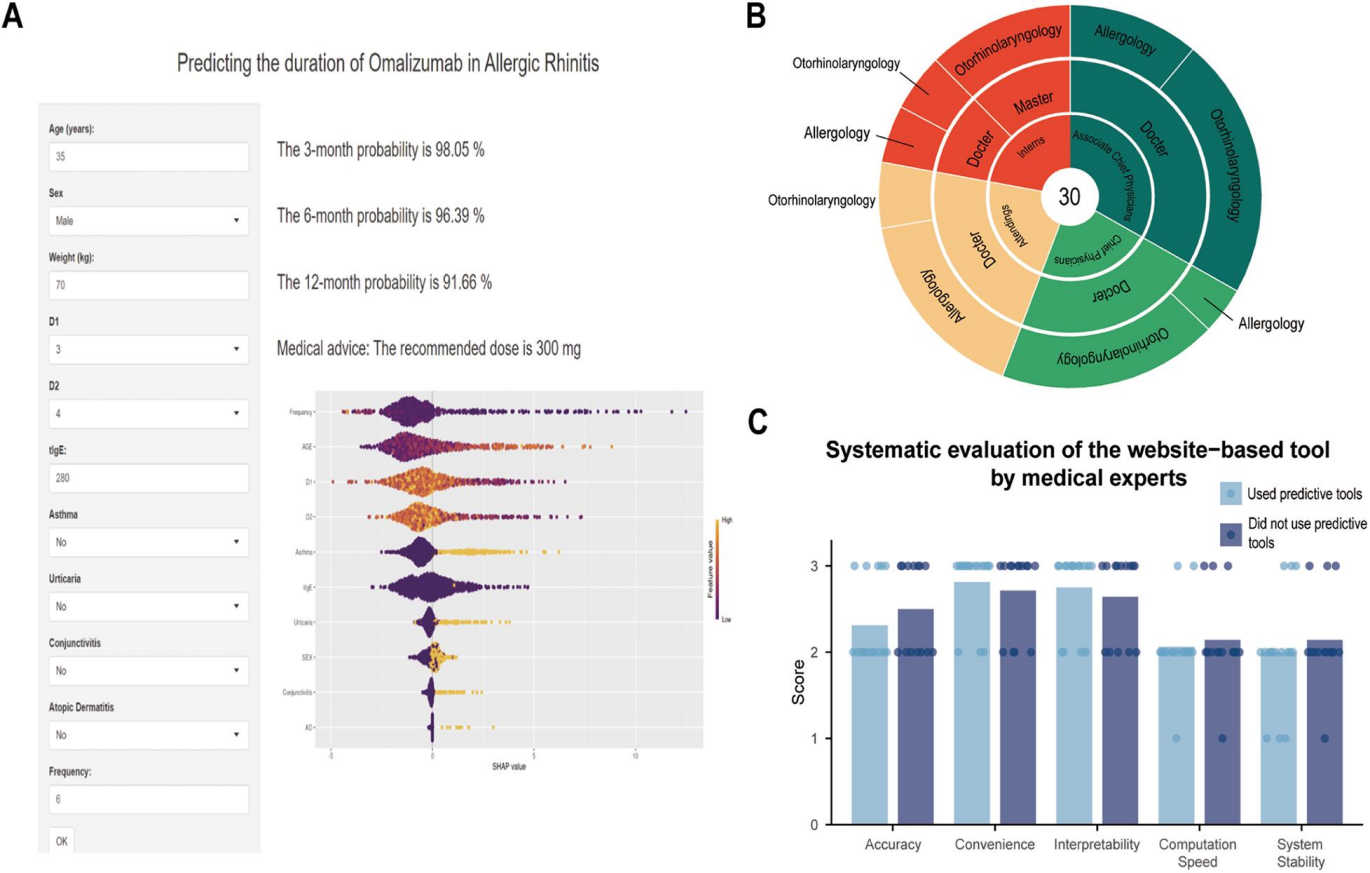
The predictive tool interface and system evaluation in actual clinical application. **(A)** The web-based calculator for predicting the efficacy duration of Omalizumab in moderate-to-severe PAR patients. **(B)** Detailed information of 30 medical experts. **(C)** Evaluation of the website-based tool by the medical experts. PAR, perennial allergic rhinitis. D1, *D. pteronyssinus*; D2, *D. farina*; tIgE: total IgE.

The final prediction model was deployed as an online web application (Figure 5A), accessible at: https://predictingthedurationofomalizumabinallergicrhinitis.shinyapps.io/Website/. By inputting values for the ten required variables, the tool automatically generated predicted Omalizumab efficacy duration for patients with moderate-to-severe PAR. Based on patient weight and tIgE concentrations, the website also calculated the recommended single dose according to existing guidelines. Figure 5B detailed information regarding physicians' clinical experience, highest educational attainment, and departmental affiliation. In real-world testing, the model demonstrated satisfactory accuracy, convenience, and interpretability, although computational speed and system stability were identified as areas for further improvement (Figure 5C).

### 3.8 Dose-response relationship

**Figure 6:**
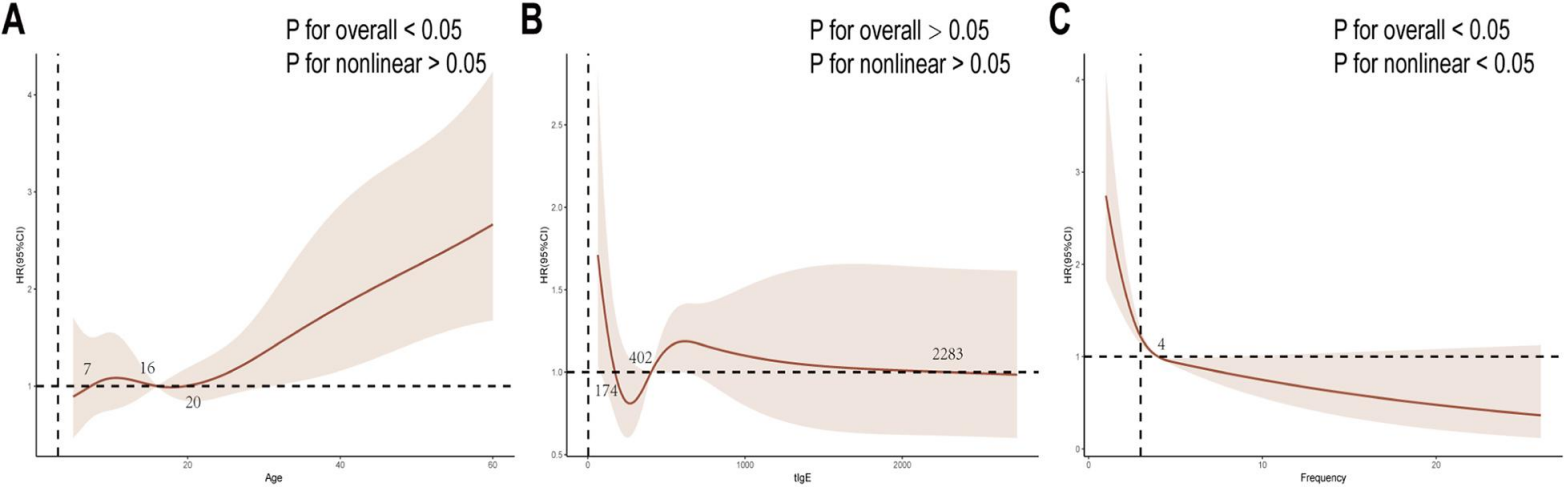
Dose-response analysis through RCS curves, including age **(A)**, tIgE **(B)**, frequency **(C)**. tIgE: total IgE.

Dose-response relationships were examined for three numerical variables: age, tIgE, and injection frequency. RCS analysis (Figure 6C) revealed a nonlinear relationship between injection frequency and outcomes (P for overall < 0.05, P for nonlinear < 0.05), with the protective effect of Omalizumab significantly increasing beyond four doses. Age exhibited a statistically linear relationship with outcomes (P for overall < 0.05, P for nonlinear > 0.05). Based on RCS analysis, statistically derived risk thresholds were identified at 7, 16, and 20 years (Figure 6A). No significant association was observed between tIgE and outcomes (P for overall > 0.05, P for nonlinear > 0.05, Figure 6B).

## 4 Discussion

Currently, the prediction of Omalizumab efficacy presents multiple clinical challenges. Most existing research has focused on asthma and CSU, with limited evidence available for AR. The clinical value of candidate biomarkers remains controversial due to inconsistent findings and practical barriers to obtaining them in routine settings. Additionally, there is a lack of predictive indicators for the duration of treatment response and applications of machine learning in this area remain limited. Therefore, this study developed and compared five machine learning models to predict the duration of Omalizumab efficacy in moderate-to-severe PAR caused by sensitization to D1 or D2, identifying key predictive factors and ultimately generating a clinically accessible web-based prediction tool.

Previous studies have explored Omalizumab efficacy prediction, but with notable limitations. In CSU, the ratio of post-treatment week 4 IgE to pre-treatment total IgE was identified as a predictor of response (16). For AR patients, research indicated that a ratio of tIgE levels at week 16 post-Omalizumab treatment to baseline levels ≥2.0 significantly correlated with clinical response to Omalizumab (22). However, these approaches require post-treatment measurements and cannot predict responsiveness prior to therapy initiation. Another study identified baseline RQLQ as predictors of Omalizumab efficacy in seasonal AR (36), but this study focused on the quality of life improvements rather than comprehensive treatment response. Elevated baseline CXCL-10 and IL-12 levels predicted favorable responses in severe asthma (21), yet cytokine testing remains costly and not routinely available. In contrast, we employed readily accessible clinical data from EMRS to construct a model that predicts duration of Omalizumab efficacy prior to treatment initiation.

Among the five machine learning models developed, RSF demonstrated favorable predictive performance. RSF is an ensemble method specifically designed for survival data, effectively handling right-censored observations without requiring linear assumptions (37). Furthermore, by constructing multiple decision trees and averaging their outputs, it mitigates the risk of overfitting associated with individual decision trees (38). Multiple studies have demonstrated the considerable value of RSF methods for predictive modelling in medical fields (39–44). In this study, we employed the RSF algorithm to establish a final model comprising ten features.

Since variables readily available in the outpatient setting were included, and the total number of variables was relatively small, variable screening was not performed. Binary data, such as gender and comorbidities, were encoded using binary encoding, which represents dichotomous variables in the most direct and efficient manner without increasing dimensionality or introducing unnecessary complexity (45).

Univariate analysis identified age, D1, D2, asthma comorbidity, and injection frequency as independent factors reflecting Omalizumab efficacy duration. Multivariate analysis did not reveal a significant effect of D2 on outcomes. Across the entire cohort, age emerged as a significant predictor (HR > 1, Supplementary Table S1, Supplementary Figure S2), consistent with previous studies identifying age as an important determinant of Omalizumab response (46). However, findings regarding age effects have been inconsistent: one study identified age over 60 years as a predictor for retreatment in CSU (47), while another observed higher recurrence rates among CSU patients under 40 years (48). Our RCS analysis revealed an S-shaped relationship between age and outcomes, with risk thresholds at 7, 16, and 20 years (Figure 6A), suggesting that adolescents (7–16 years) and adults (over 20 years) may face higher recurrence risk after treatment discontinuation. Notably, this finding was derived from real-world data with a relatively small sample size, and thus may be affected by potential selection bias. However, Foti et al. observed no significant age difference between recurrent and non-recurrent patients with CSU following Omalizumab treatment (49).

We found that D1 and D2 were protective factors in univariate Cox analysis (HR < 1, *P* < 0.05, Supplementary Table S1), indicating that patients with higher dust mite sensitization levels experienced stronger protective effects from Omalizumab. This aligns with previous work showing that airborne allergen sensitization predicted Omalizumab responsiveness in children with persistent autumn asthma attacks (50).

In our study, injection frequency also demonstrated a protective effect (HR < 1, *P* < 0.05, Supplementary Table S1, Supplementary Figure S2), with dose-response analysis revealing significantly enhanced protection beyond four injections (Figure 6C). This conclusion aligned with prior research. Ceravalls et al. found symptom recurrence in CSU patients was significantly correlated with Omalizumab treatment duration, with a threshold of 6.5 months (51). However, the observed better outcomes after the fourth injection should be interpreted cautiously, as this pattern may reflect potential selective bias of responders rather than a strict causal effect of the number of injections alone.

Currently, the role of tIgE in predicting Omalizumab efficacy remains debated. In this study, neither univariate (Supplementary Table S1) nor multivariate Cox analysis (Supplementary Figure S2), nor dose-response relationship analysis (Figure 6B), demonstrated a significant association between tIgE and the duration of Omalizumab treatment efficacy for moderate-to-severe PAR (*P* > 0.05). This aligns with findings from a large Italian CSU cohort, where tIgE correlated with initial response but not with recurrence timing (52). However, conflicting evidence exists: some studies reported that tIgE > 100 kU/L was associated with accelerated CSU recurrence after Omalizumab discontinuation (53), and higher baseline tIgE levels were observed in asthma patients requiring retreatment (54). These discrepancies may reflect disease-specific differences or varying patient populations.

A key strength of this study lies in its methodological rigor. Data were drawn from three distinct healthcare institutions, enhancing model robustness and generalizability. We employed repeated ten-fold cross-validation with 400 iterations to obtain stable performance estimates and avoid overfitting to specific data splits (55, 56). The RSF model was selected based on comprehensive evaluation across training, internal test, and external test sets, considering both discriminative ability and calibration.

The utilization of machine learning-based methods was dictated by the heterogeneity of moderate-to-severe PAR patients responding to Omalizumab. By employing SHAP to interpret the model, we can identify the most influential variables (Figure 3). The SHAP methodology elucidates the model's functionality through global and local explanations, detailing how personalized input data leads to specific predictions for individual patients. This transparency is crucial for fostering clinician trust and facilitating adoption in clinical practice. The top five predictors—injection frequency, age, D1, D2, and comorbid asthma—represent routinely collected clinical variables, eliminating the need for additional testing. Furthermore, the final model was deployed as an openly accessible web-based tool (https://predictingthedurationofomalizumabinallergicrhinitis.shinyapps.io/Website/). This user-friendly interface bridges the gap between predictive analytics and real-world patient management. Thirty medical experts with diverse backgrounds evaluated the tool, confirming satisfactory accuracy, convenience, and interpretability, while identifying computational speed and system stability as areas for future refinement.

However, our study also has certain limitations. First, its retrospective design introduces potential recall bias, and dose adjustments of background medications during follow-up may have influenced efficacy duration assessments. Second, the findings are derived from a Chinese population sensitized to D1 or D2, limiting generalizability to other geographic regions or ethnic groups with different genetic backgrounds, environmental exposures, and practice patterns. Third, we focused on clinical biomarkers without incorporating socioeconomic factors such as insurance coverage, household income, or educational attainment. Given the high cost of Omalizumab treatment, patients' financial capacity and early therapeutic response to Omalizumab may substantially impact treatment adherence and persistence. Fourth, although type 2 inflammatory markers such as eosinophil counts and fractionated exhaled nitric oxide (FeNO) are routinely available, their limited specificity and susceptibility to medication effects led to their exclusion; however, these data are being collected for future larger-scale, multinational studies. Fifth, the model predicts the probability of sustained efficacy over specific timeframes rather than providing exact durations for individual patients. Future work with larger samples and advanced techniques may address these limitations. Despite these limitations, the outstanding performance of our final predictive model is not obscured.

In summary, we successfully developed an interpretable machine learning model to predict the duration of efficacy in moderate-to-severe PAR patients treated with Omalizumab, based on readily accessible outpatient clinical data. The final RSF model demonstrated excellent predictive performance across internal and external test sets, and its deployment as a web-based tool facilitates clinical application. These findings provide a valuable resource for personalized treatment decisions and highlight key factors affecting the durability of Omalizumab response.

## Data Availability

The original contributions presented in the study are included in the article/Supplementary Material, further inquiries can be directed to the corresponding author/s.
