# Supplementary material for: Toward personalized prediction: a multicenter machine learning model for omalizumab response duration in moderate-to-severe perennial allergic rhinitis
Source: Front Allergy. 2026 Apr 10;7:1784032. doi: 10.3389/falgy.2026.1784032 (PMC13106427; doi:10.3389/falgy.2026.1784032)
Supplement: Supplementary file 1 [file Table1.docx]

Supplementary Material

# Supplementary Figures

##
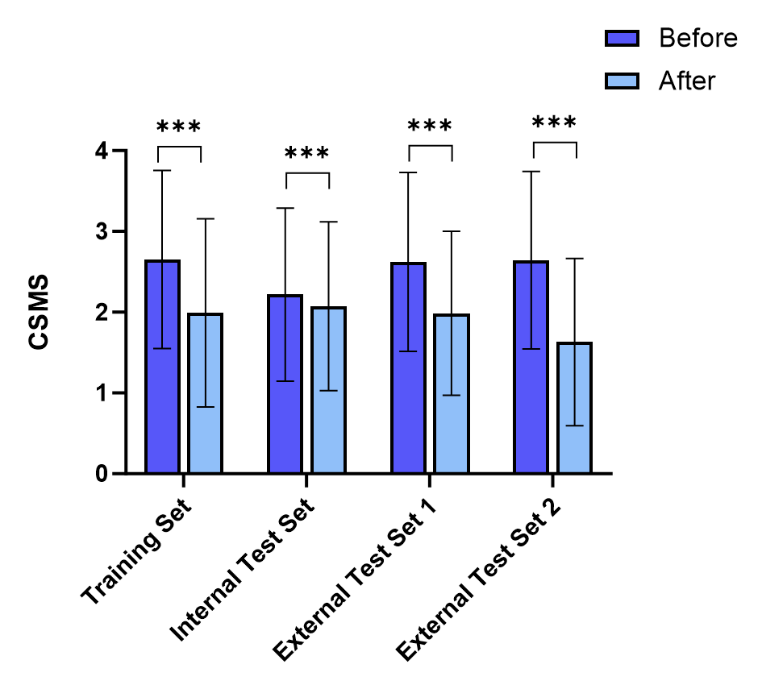
Fig.S1

Fig.S1 Bar chart showing the changes in CSMS before and after Omalizumab treatment in different datasets. CSMS, combined symptom and medication score. * P<0.05; ** P<0.005; *** P<0.001.

##
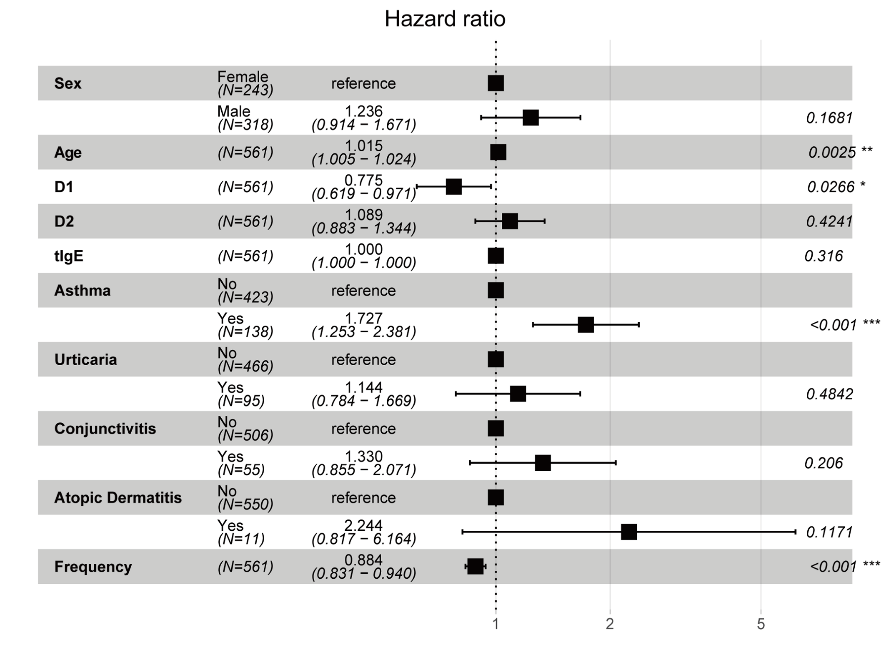
Fig.S2

Fig.S2 Forest plot of multivariate Cox regression analysis based on the entire cohort. D1, *D. pteronyssinus*; D2, *D. farina*; tIgE: total IgE. *P<0.05; **P<0.005; ***P<0.001.

## Fig.S3


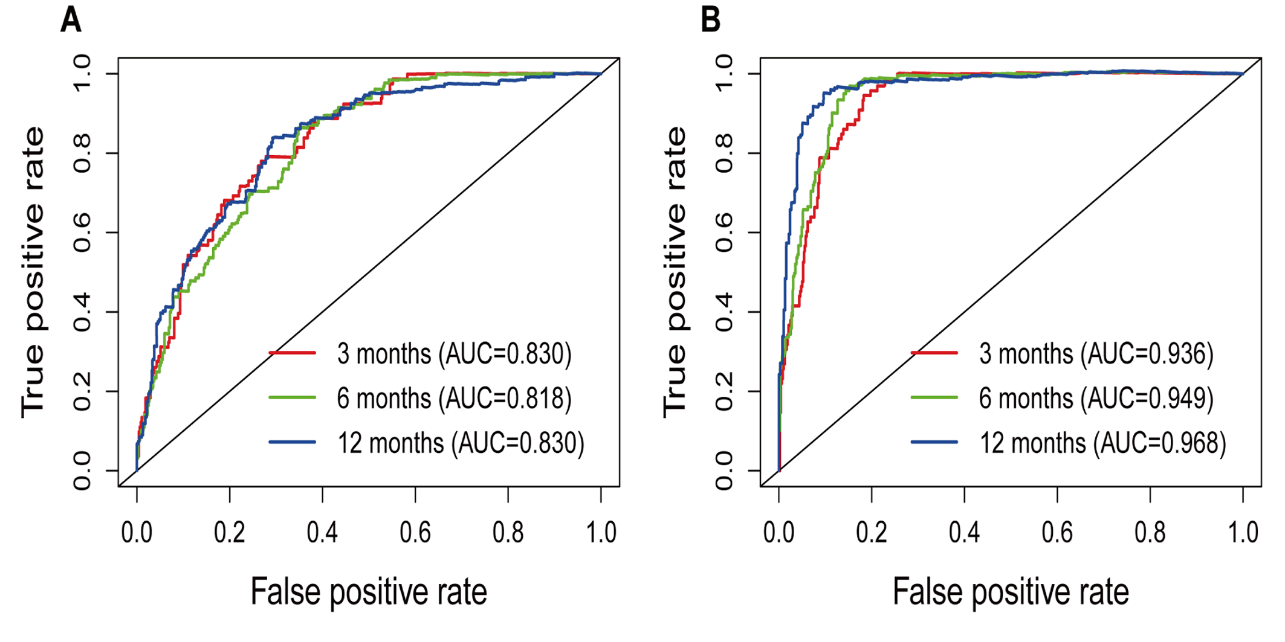


Fig.S3 Evaluation of performance: before(A) versus after(B) the implementation of RSF optimization. RSF: random survival forest.

# Supplementary Tables

## Table S1

## Table S1 Univariate Cox regression analysis based on the entire cohort.

Abbreviations: D1, *D. pteronyssinus*; D2, *D. farina*; tIgE: total IgE. *P<0.05

## Table S2

Table S2 The detailed characteristics of the example patients.

Abbreviations: D1, *D. pteronyssinus*; D2, *D. farina*; tIgE: total IgE. AD, atopic dermatitis.
